# Supplementary material for: Single nucleosome imaging reveals principles of transient multiscale chromatin reorganization triggered by histone ADP-ribosylation at DNA lesions
Source: Nat Commun. 2025 Jul 19;16:6652. doi: 10.1038/s41467-025-61834-7 (PMC12276361; doi:10.1038/s41467-025-61834-7)
Supplement: Supplementary file 1 — Supplementary Information [file 41467_2025_61834_MOESM1_ESM.pdf]

# Supplementary information

## Garcia-Fernandez *et al*.

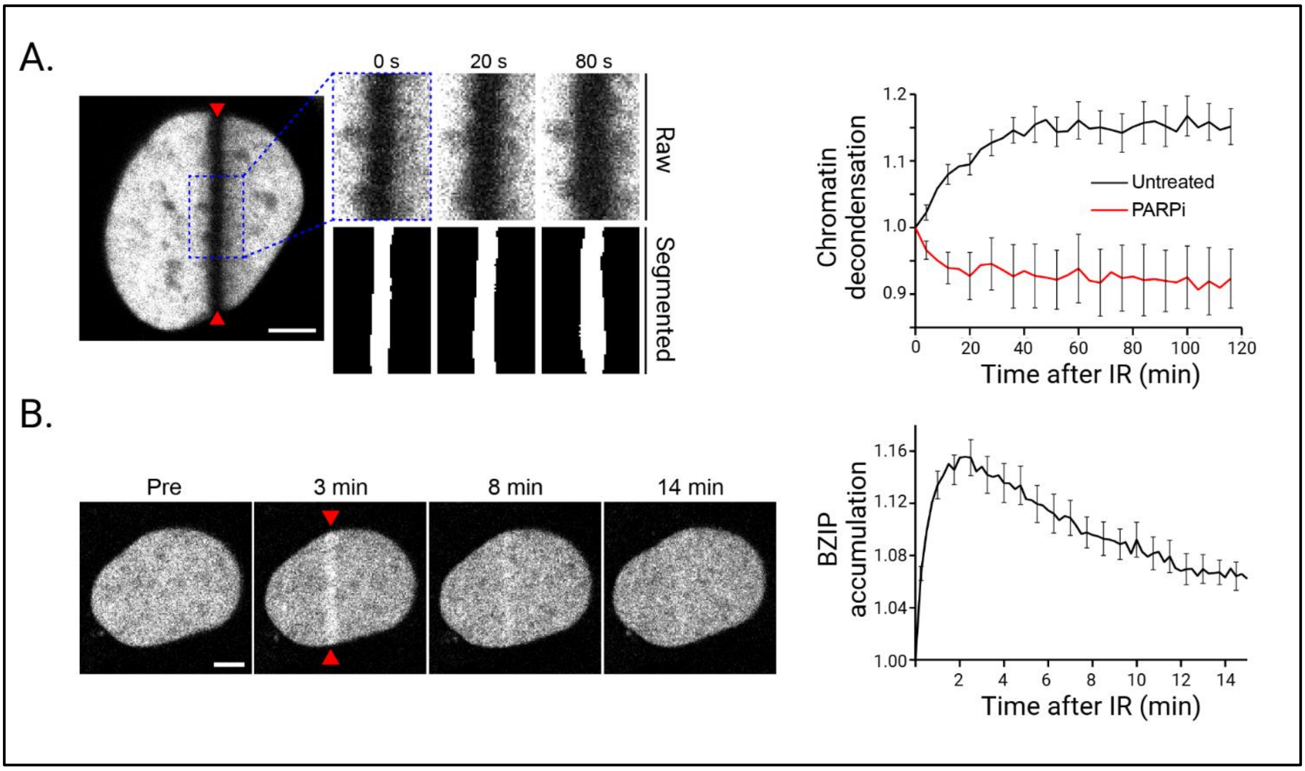


**Supplementary Figure 1: Chromatin decondensation and BZIP accumulation at DNA lesions.** (A) The irradiation of the nucleus of a U2OS cell expressing H2B-EGFP with a pulsed laser at 355 nm triggers both DNA damage and photobleaching of the irradiated region (red arrowheads). After segmentation of the photobleached line, its thickness is plotted as a function of time after irradiation and normalized to time zero to estimate the changes in the overall chromatin compaction state. The graph on the right shows the line thickness (mean ± SEM) for cells left untreated (n=15) or treated with 30 µM of the PARP inhibitor Olaparib (n=15). (B). Confocal image sequence of the nucleus of U2OS cells expressing the DNA binding domain BZIP fused to EGFP, used as a sensor of DNA accessibility. The cell was presensitized with Hoechst and irradiated with a 405 nm laser (red arrowheads). The graph on the right shows the BZIP recruitment kinetics obtained from the confocal images (mean ± SEM, n=18). All scale bars: 4 µm. Created in BioRender. Garcia Fernandez, F. (2025) https://BioRender.com/5akfy1x

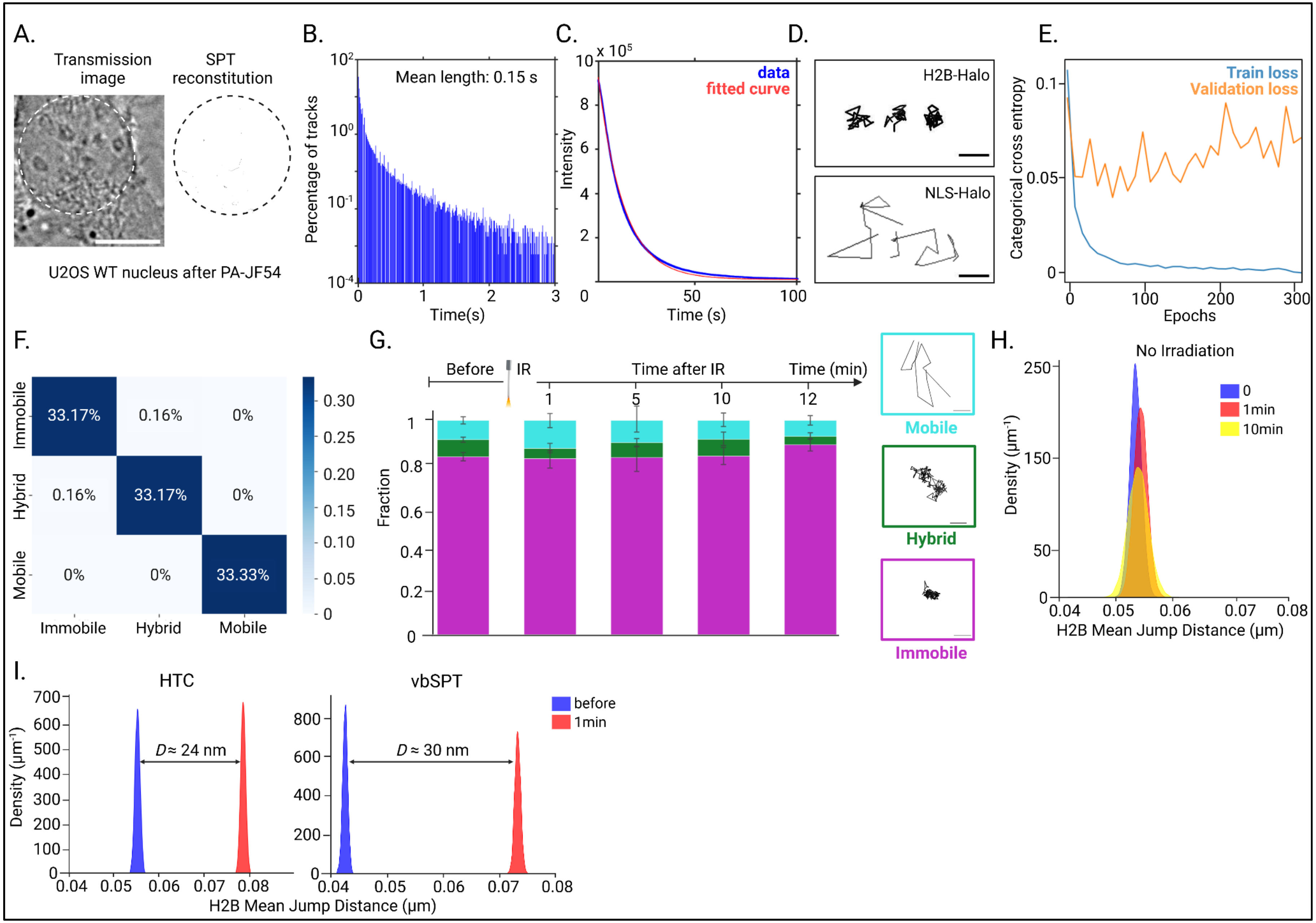

A.
Transmission image
SPT reconstitution
U2OS WT nucleus after PA-JF54
B.
Mean length: 0.15 s
Percentage of tracks
Time(s)
C.
x 10^5
Intensity
data
fitted curve
Time (s)
D.
H2B-Halo
NLS-Halo
E.
Train loss
Validation loss
Categorical cross entropy
Epochs
F.
Immobile
Hybrid
Mobile
33.17%
0.16%
0%
0.16%
33.17%
0%
0%
0%
33.33%
G.
Before
IR
Time after IR
Time (min)
Fraction
Mobile
Hybrid
Immobile
H.
No Irradiation
0
1min
10min
Density (µm⁻¹)
H2B Mean Jump Distance (µm)
I.
HTC
vbSPT
D≈ 24 nm
D ≈ 30 nm
before
1min
Density (µm⁻¹)
H2B Mean Jump Distance (µm)

**Supplementary Figure 2. Tracking of single fluorescently tagged H2B proteins in living cells.** (A) Control U2OS cell that does not express H2B-Halo after tagging with PA-JF549 Halo ligand. The widefield transmission image of the nucleus (highlighted by the dotted line) is shown on the left and the single molecule trajectories that were detected is shown on the right. (B) Distribution of track lengths for U2OS cells expressing H2B-Halo and tagged with PA-JF549 Halo ligand in the absence of DNA damage. The histogram combines 60 cells representing 68779 trajectories (mean length of 0.17s). (C) Characteristic bleaching curves for PA-JF549 Halo ligand tagging H2B-Halo expressed in U2OS cells. The experimental data (blue) were fitted with the single exponential decay (red) to recover a characteristic bleaching time of 12.3 s (n=5). (D) Representative single molecule trajectories for H2B-Halo (top) and NLS-Halo (bottom) tagged with PA-JF549 Halo ligand. (E) Training and validation cross entropy losses for the CNN model used to classify the single molecule H2B trajectories. Minimum for the validation set is reached at 6 epochs. (F) Confusion matrix of the trained model on the validation set of H2B trajectories. (G) Distribution of the H2B trajectories obtained from the CNN classifier at different time points after irradiation at 355 nm. Magenta, green and cyan bars represent immobile, hybrid and mobile H2B populations, respectively. Representative examples of trajectories are shown on the right. Scale bars: 100 nm. (H) Mean jump distance KDE plots for the immobile population of H2B tracks in the absence of micro-irradiation. Number of cells analyzed (N): $N_{bef}$=4, 96 trajectories; $N_{1min}$=4, 181 trajectories; $N_{10min}$=4, 189 trajectories. (I) Mean jump distance KDE plots for the immobile population of H2B tracks before and 1 minute after micro-irradiation analyzed by the HTC (right) and vbSPT (left) softwares. Created in BioRender. Garcia Fernandez, F. (2025) https://BioRender.com/5akfy1x

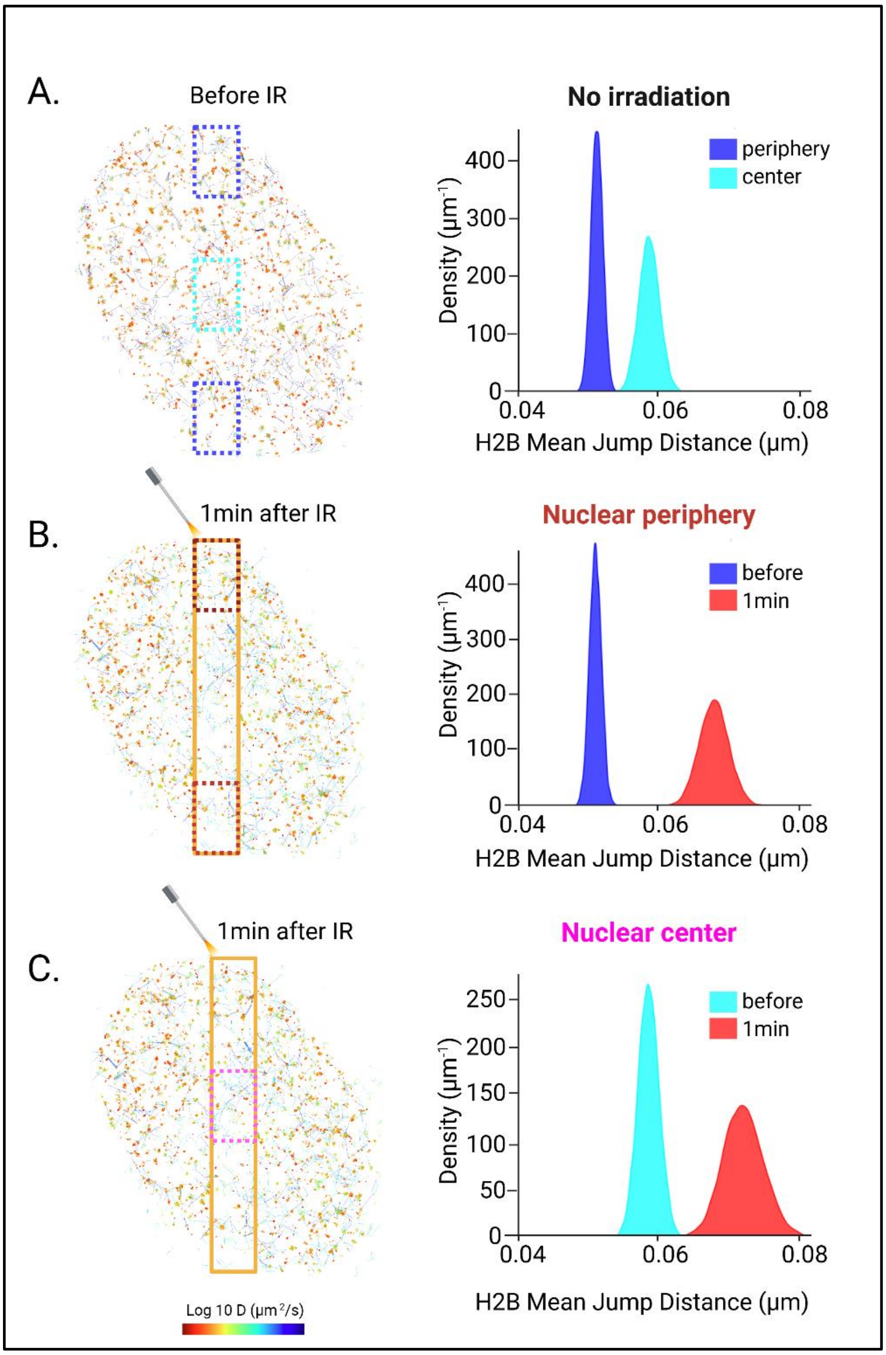


**Supplementary Figure 3. Assessing H2B dynamics depending on the distance to the nuclear rim.** Left, trajectories of individual histones in the nucleus of a U2OS cell expressing H2B-Halo bound to PA-JF549 Halo ligand. H2B motions were monitored at the nuclear periphery (blue boxes) and nuclear center (cyan box) in the absence of damage (A) and 1 minute after irradiation (red and magenta boxes in B and C, respectively). The trajectories are color-coded according to their diffusion coefficient using the look-up table shown below. Right, mean jump distance KDE plots for the immobile population of H2B tracks for each condition. Number of cells analyzed (N). Nuclear periphery: $N_{bef}$=10, 301 trajectories; $N_{1min}$=10, 91 trajectories; Nuclear center: $N_{bef}$=8, 201 trajectories; $N_{1min}$=8, 47 trajectories. Created in BioRender. Garcia Fernandez, F. (2025) https://BioRender.com/5akfy1x

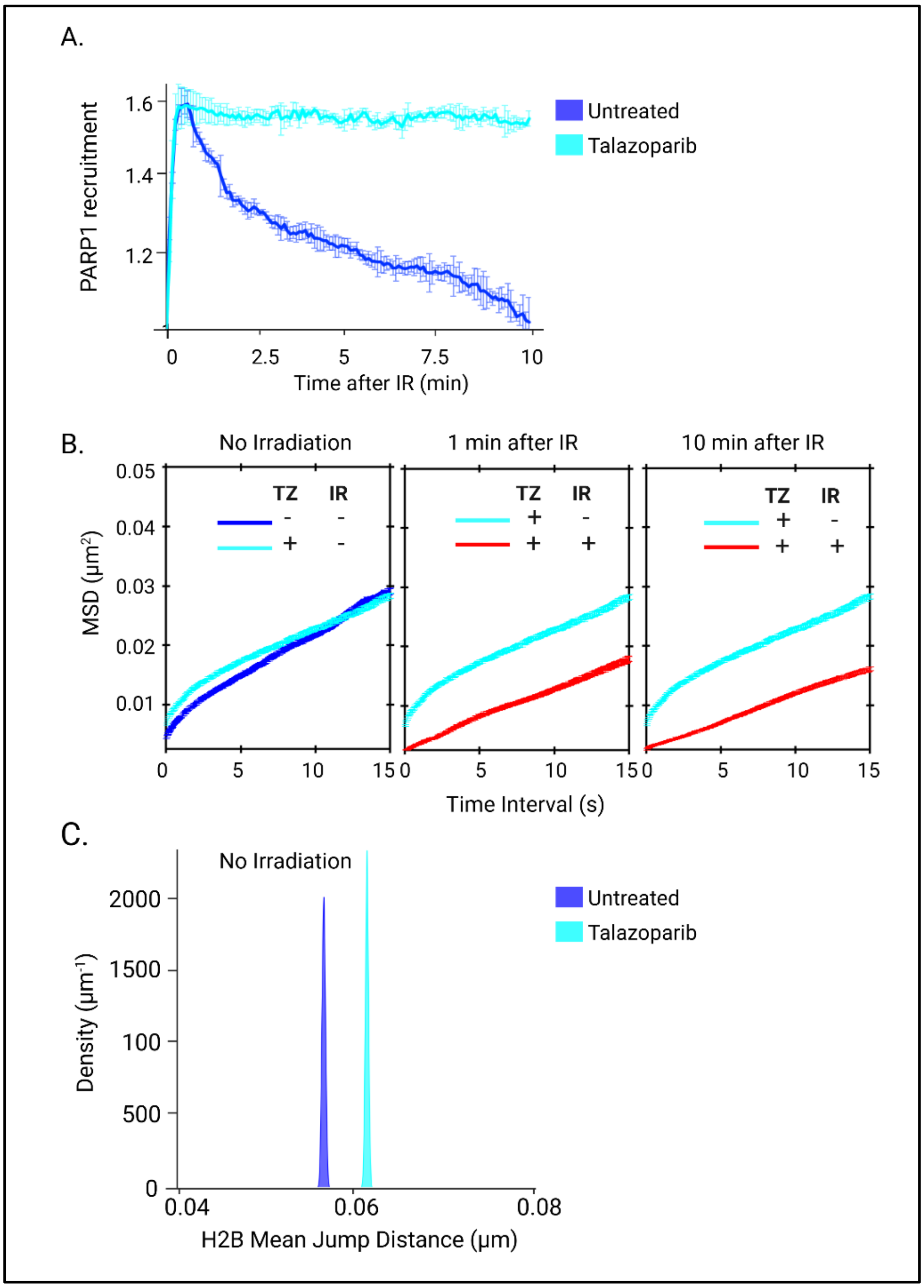


**Supplementary Figure 4. Impact of PARP inhibition on chromatin dynamics.** (A) Kinetics of PARP1 recruitment to DNA damage induced by 355 nm irradiation in PARP1 KO U2OS cells transiently transfected with EGFP-PARP1, left untreated or treated with 30 µM of Talazoparib (N=10 for each condition). Recruitment was estimated by measuring the mean fluorescence intensity within the manually segmented irradiated region. This fluorescence signal was background subtracted and corrected for photobleaching by dividing it to the mean intensity of the whole nucleus. This corrected intensity was then normalized to the value prior to damage. (B) Mean squared displacement curves of the fluorescently tagged *lacO* array in cells

treated or not with 30 µM Talazoparib, before, 1 min and 10 min after irradiation (N=10). (C) H2B mean jump distance histograms for the immobile population of H2B tracks in WT cells treated or not with 30 µM Talazoparib in the absence of DNA damage (untreated N=35, 1376 trajectories; Talazoparib N=42, 15672 trajectories). Mean jump distance between Talazorib-treated condition *versus* untreated condition is significantly different ($p < 0.001$, calculated from Yuen-Welch Test). Created in BioRender. Garcia Fernandez, F. (2025) https://BioRender.com/5akfy1x

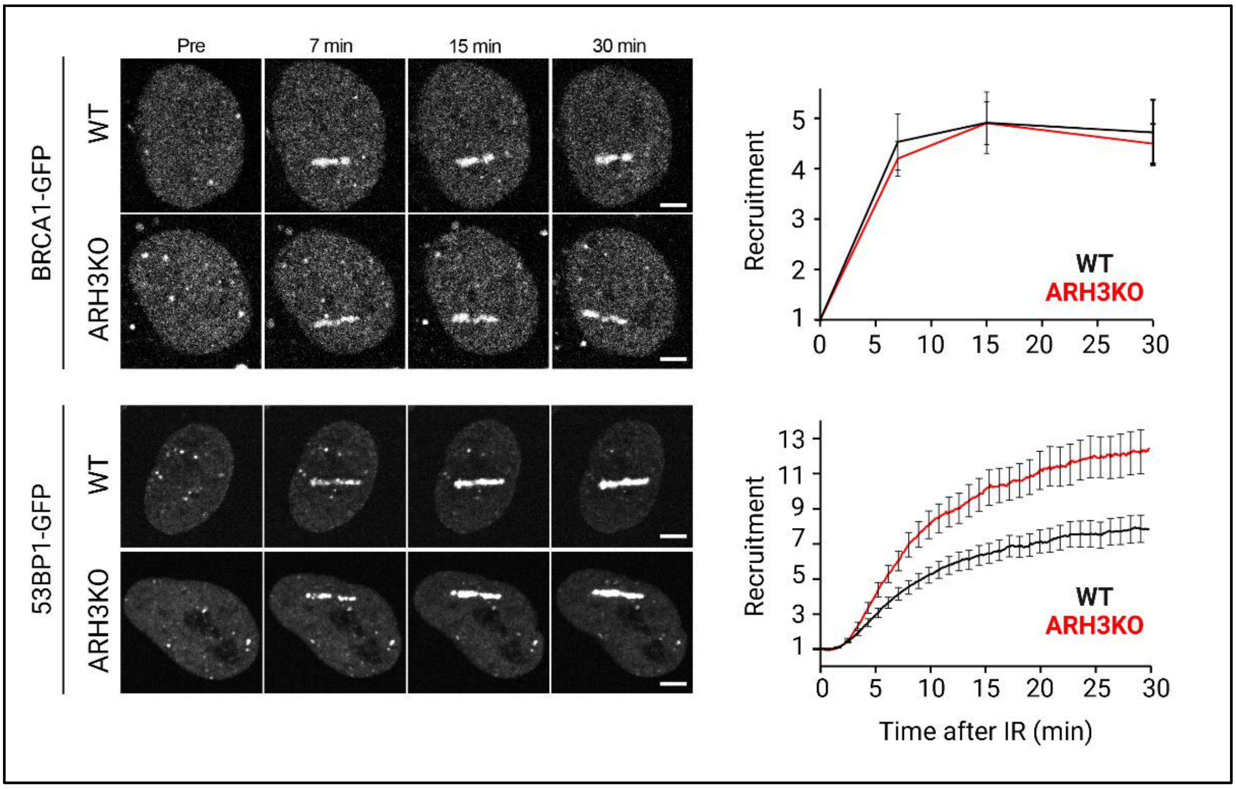


**Supplementary Figure 5. Impact of the loss of ARH3 on the recruitment of repair factors.** Confocal image sequence and recruitment kinetics of BRCA1-GFP and 53BP1-GFP in WT and ARH3 KO U2OS cells after irradiation at 800 nm or 405 nm, respectively. Scale bars: 4 µm. Data are shown as mean ± SEM. (BRCA $N_{WT}$=30, $N_{ARH3}$=32; 53BP1 $N_{WT}$=18, $N_{ARH3}$=18). Created in BioRender. Garcia Fernandez, F. (2025) https://BioRender.com/5akfy1x

| Softwares | Parameters | values |
|---|---|---|
| **Slimfast** | Localization error<br>Deflation loops<br>Max OFF time<br>Max D<br><br>Number minimum of points in a trajectory | $10^{-6}$<br>0<br>1<br>7 $\mu m^2/s$<br><br>2 |
| **vbSPT** | Number of hidden states<br>Time step<br>Initial D<br>Bootstrap number<br>Convergence criterion<br>Prior type<br>Prior D strength<br>Prior type Pi<br>Prior piStrength<br><br>Other input parameters followed the suggestion of the vbSPT authors | 2<br>0.01<br>0.001 and 2<br>100<br>$10^{-8}$<br>mean strength<br>5<br>Natmet13<br>5 |
| **HTC** | Minimum length of immobile trajectory considered for the analysis<br><br>Bandwidth factor for the Gaussian kernel in KDE(Kernel density estimation) plot<br><br>Resampling number for the bootstrapping<br><br>Trim value for the Yuen-Welch's t-test<br><br>Confidence level of bootstrapping | 8 frames /80ms<br><br>1<br><br>10000<br><br>0.1 (10% for both sides)<br><br>0.95 |

**Supplementary Table. Parameter values for the different softwares used to track and analyze histone dynamics.**
