# Supplementary material for: Single nucleosome imaging reveals principles of transient multiscale chromatin reorganization triggered by histone ADP-ribosylation at DNA lesions
Source: Nat Commun. 2025 Jul 19;16:6652. doi: 10.1038/s41467-025-61834-7 (PMC12276361; doi:10.1038/s41467-025-61834-7)
Supplement: Supplementary file 3 — Description of Additional Supplementary Files [file 41467_2025_61834_MOESM3_ESM.pdf]

Description of Additional Supplementary Files

File Name: Supplementary Movie 1

Description: Typical U2OS nucleus expressing H2B fused to Halo and labelled to PAJF549. Acquisition time: 10ms. Colored lines represent trajectories of individual histones.
